# Effect of population structure and kinship relationships on the results of association mapping tests of growth and wood quality traits in four *Eucalyptus* populations

**DOI:** 10.1186/1753-6561-5-S7-P23

**Published:** 2011-09-13

**Authors:** Eduardo Pablo Cappa, Maria C Martínez, Martín N Garcia, Pamela V Villalba, Susana N Marcucci Poltri

**Affiliations:** 1 Instituto Nacional de Tecnología Agropecuaria. Instituto de Recursos Biológicos. Hurlingham, Buenos Aires, Argentina; 2 Instituto Nacional de Tecnología Agropecuaria. Instituto de Biotecnología. Hurlingham, Buenos Aires, Argentina

## Background

In recent years, association mapping studies have been reported for growth and wood quality traits in *Eucalyptus* (e.g. [1]). One problem with association studies is that they can be sensitive to the presence of population structure. The presence of population structure may generate spurious associations between markers and traits and leading to an elevated false-positive rate (e.g. [2]). Statistical approaches that account for population structure include model-based clustering [3], principal component analysis, genomic control and linear mixed model approach [4]. The mixed model of Yu et al. (2006) accounts for both major population structure, assigning individuals to subpopulations (the Q matrix), and the relatedness among individuals within and between subpopulations (the kinship (K) matrix). The mixed model approach generally performs best [2,4].

As part of the Biotech MERCOSUR project (Marcucci Poltri et al. this volume) molecular and phenotypic data from four *Eucalyptus* populations have been obtained: three open pollinated (OP, half-sib) progeny trials of *Eucalyptus grandis* from Argentina (EgrAr) and *Eucalyptus globulus* from Uruguay (EglUy) and Argentina (EglAr) and one clonal trial of *Eucalyptus grandis* from Paraguay (EgrPy). These populations differ in the underlying substructure and genetic relatedness among individuals. It is thus important to investigate the effects of population structure and kinship on the results of associations between markers and growth and wood quality traits from these *Eucalyptus* populations.

## Material and methods

A total of 612 trees were sampled from the EgrAr (188), EgrPy (121), EglAr (134) and EglUy (169) populations. The number of OP families sampled in each OP progeny trials was 132 (EgrAr), 129 (EglAr) and 70 (EglUy) from different native stand sites in Australia (from 8 to 13) and land races (from 1 to 3). The number of trees per OP family varied from 1 to 3 (EgrAr), 1 to 8 (EglUy) and 1 to 2 (EglAr). One growth trait (diameter at breast height, DBH) and three quality wood traits (extractives in ethanol, Klason lignin and syringyl:guaiacyl ratio (S:G ratio)) were studied.

All the 612 trees were genotyped using Diversity Arrays Technology (DArT) molecular markers [5]. A subset of 2816 (EgrAr), 2693 (EgrPy), 2373 (EglAr) and 2300 (EglUy) DArT markers were used in the analysis after markers with frequency greater than 0.95 or less than 0.05 were excluded.

The association mapping tests were carried out at the DArTs level using two-steps. First, for the three OP trials, the overall mean and design effects or first order autoregressive residuals for rows and columns, were fitted to deal with environmental variation. Additionally, for the clonal trial, the best linear unbiased predictions (BLUP) of clonal values were predicted. Second, the markers effects were tested on the adjusted phenotypes (OP trials) or clonal BLUPs values (clonal trial) using four models [4]: 1) Simple model, in which Q and K matrices are ignored; 2) Q model, considers only Q matrix; 3) K model, considers only K matrix; and 4) Q+K model, considers both Q and K matrices. Except for the EglAr population, the Q matrix was calculated by the software STRUCTURE [3] on basis of 400 random DArT markers. The K matrix was calculated on basis of 800 random DArT markers using the software package SPAGeDi [6].

For each of the four populations, all the association tests were carried out using TASSEL software [http://www.maizegenetics.net/].

## Results

All populations showed an optimum cluster number of 4. In general, for the three OP populations the compositions of the cluster coincide with the geographical native stand sites in Australia. More than 52% of the pair-wise kinship estimates were equal to 0, whereas about the 46% of the values were less than 0.25. Without taking into account the population structure and kinship (Simple model), from 0.9 to 8.5 % of the DArT markers tested were associated with the growth and wood quality traits at *P* < 0.01. These preliminary results show a high number of associated markers that might suggest that several of them are likely to be false-positives due to population structure and/or kinship relationships among trees within each population. For all populations and most traits under consideration, the controlling only for population structure (Q model) reduced the number of significant DArTs (from 0.5 to 2.6%). This effect was more pronounced for the two OP *Eucalyptus globulus* populations. However, when the relative kinship coefficients between every pair of individuals were considered (K model), a more stringent reduction with respect to the Q model was observed (from 0.3 to 1.3%). This finding suggests that, when more complex interrelationship among individuals within and between subpopulations exists, the fitting Q model was not enough to reduce the number of spurious associations. It is no clear, across populations and traits, that the Q matrix should be added to the kinship effect. The reduction of number of significant DArT markers, excluding pedigree information (K matrix) in the model, appeared to be trait dependent.

## Conclusions

Both population structure and kinship relatedness between individuals had effect on the association mapping tests. The kinship had a more stringent effect on the marker-trait associations. Effect on association tests depends on the populations and traits studied.
